# Anti-Inflammatory and Antioxidant Chinese Herbal Medicines: Links between Traditional Characters and the Skin Lipoperoxidation “Western” Model

**DOI:** 10.3390/antiox11040611

**Published:** 2022-03-23

**Authors:** Jose M. Prieto, Guillermo R. Schinella

**Affiliations:** 1 School of Pharmacy and Biomolecular Sciences, Liverpool John Moores University, Liverpool L3 3AF, UK; 2 Facultad de Ciencias Médicas, Universidad Nacional de La Plata, La Plata B1900, Argentina; schinell@med.unlp.edu.ar; 3 Instituto de Ciencias de la Salud, UNAJ-CICPBA, Florencio Varela BA1888, Argentina

**Keywords:** traditional Chinese medicine, phytotherapy, skin inflammation, lipoperoxidation, eicosanoids, antioxidants

## Abstract

The relationship between lipid peroxidation and inflammation has been accepted as a paradigm in the field of topical inflammation. The underlying biochemical mechanisms may be summarised as unspecific oxidative damage followed by specific oxidative processes as the physio pathological response in skin tissues. In this experimental review we hypothesise that the characteristics attributed by Traditional Chinese Medicine (TCM) to herbal drugs can be linked to their biomolecular activities within the framework of the above paradigm. To this end, we review and collect experimental data from several TCM herbal drugs to create 2D-3D pharmacological and biochemical spaces that are further reduced to a bidimensional combined space. When multivariate analysis is applied to the latter, it unveils a series of links between TCM herbal characters and the skin lipoperoxidation “Western” model. With the help of these patterns and a focused review on their chemical, pharmacological and antioxidant properties we show that cleansing herbs of bitter and cold nature acting through removal of toxins—including *P. amurense*, *Coptis chinensis*, *S. baicalensis* and *F. suspensa*—are highly correlated with strong inhibition of both lipid peroxidation and eicosanoids production. Sweet drugs—such as *A. membranaceus*, *A. sinensis* and *P. cocos*—act through a specific inhibition of the eicosanoids production. The therapeutic value of the remaining drugs—with low antioxidant or anti-inflammatory activity—seems to be based on their actions on the Qi with the exception of furanocoumarin containing herbs—*A. dahurica* and *A. pubescens*—which “expel wind”. A further observation from our results is that the drugs present in the highly active “Cleansing herbs” cluster are commonly used and may be interchangeable. Our work may pave the way to a translation between two medical systems with radically different philosophies and help the prioritisation of active ingredients with specific biomolecular activities of interest for the treatment of skin conditions.

## 1. Introduction

Topical inflammation underpins almost every skin condition. The search for new, safer therapies to both acute skin conditions such as mechanical injuries and UV exposition and chronic conditions such as eczema, psoriasis, and atopic dermatitis, among many other skin pathologies, is therefore warranted.

The pathophysiological mechanisms of chronic topical inflammatory conditions such as atopic dermatitis and psoriasis are complex. Much emphasis has been put on the extremely complex interplay between the skin and immune system in terms of inflammatory mediators. Therefore, old and current medical approaches favour aggressive anti-inflammatory, immunosuppresive [1] and photoactive [2] drugs (steroids, furanocoumarins, cyclosporine, etc.) with a poor balance between pharmacological and toxicological effects in the long term. Although it is well-recognized that many of these conditions are accompanied by a burst of free radicals and imbalanced antioxidant defences both at both local and systemic levels, antioxidant therapies are yet to be fulfilled [3,4,5]. This is due to a lack of clear target, given the enormous array of chemical species and secondary mediators involved in the cell redox biology [6].

### 1.1. Introduction to the “Western” Skin Lipoperoxidation Model

The concept of lipid peroxidation as a central pathophysiological process in skin diseases has gained the attention of a part of the research community since the 1980s. The relationship between lipid peroxidation and skin inflammation has been perfectly laid out by Briganti and Picardi [7] and De Luca and Valacchi [8], whilst the underlying biochemical mechanisms were thoroughly reviewed by Guteridge [9] and Niki [10].

**Figure 1:**
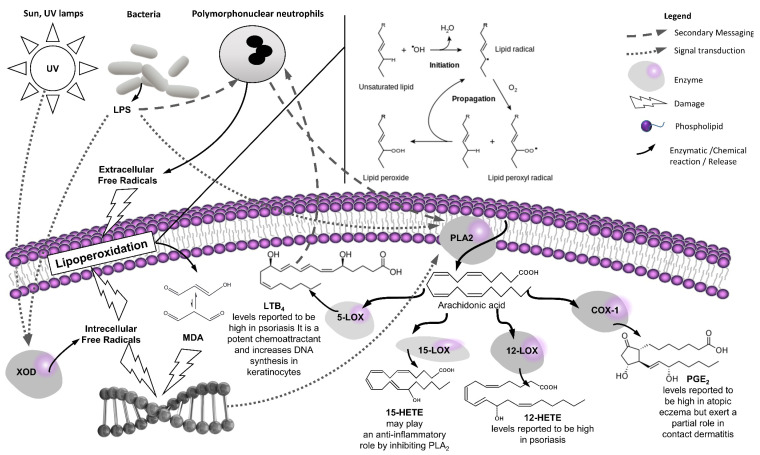
Relationship between lipoperoxidation and skin inflammatory conditions.

Within the theoretical framework laid out by these authors we want to focus on two aspects within the multifaceted and complex interplay between lipoperoxidation and skin inflammation that are pertinent to our paper: the unspecific peroxidation of membrane lipids as a damaging factor [11] and the specific peroxidation of certain membrane lipids (arachidonic acid chiefly) as a response against this damage. The first process physically destabilizes the cell function by altering the structure of its membranes and generates toxic end products such as malonyl dialdehyde (MDA) [12]. The second one generates a family of secondary pro- [13] and anti-inflammatory lipid mediators [14] known as eicosanoids. The most important eicosanoids, prostaglandins and leukotrienes, are biosynthesised from arachidonic acid and are responsible in the early stages of the inflammatory process for the attraction of neutrophils to the affected tissue. On the one hand, high levels of these mediators maintained over time will contribute to a chronic condition [15]. On the other hand, their continuous biosynthesis may also help the resolution of the inflammatory process as they promote the induction of 15-lipoxygenases necessary for the biosynthesis of lipoxin, derived from the ω-6 fatty acid arachidonic acid, and resorbin, protectin, and maresin, derived from the ω-3 fatty acids eicosapentaenoic acid and docosahexaenoic acid, with many of them described as being synthesised by skin cells [16,17]. However, the activity of traditional herbal medicines on these anti-inflammatory mediators is just starting to be scrutinised [18], and therefore we will focus on proinflammatory eicosanoids to study the relationship of curative effects of such herbal drugs with their traditional characters in acute inflammation. Their interplay and actions are summarized in Figure 1.

The inhibition of lipid peroxidation may be a therapeutic target [7]. However, unspecific inhibitors of lipid peroxidation may successfully quench the free radicals and stop the chain reaction leading to lipid peroxides and MDA but could theoretically at the same time impair the synthesis of eicosanoids, which may promote wound healing in the early phases of skin damage. Therefore, a more rational therapeutic approach to this conundrum would be the use of specific inhibitors of the different types of lipoperoxidation in different ratios depending on the stage of the condition. Modern medicine has developed NSAIDs to specifically inhibit eicosanoids production and steroids to reduce the expression of the enzymes involved in their biosynthesis. Yet there is not a defined clinical approach to specifically quench the radicals and stop membrane lipids peroxidation [19].

### 1.2. Introduction to the “Eastern” Skin Inflammation Model

All cultures have developed over millennia therapeutic approaches to skin conditions. This is not surprising, considering that the skin is the most accessible and largest “organ”. Among these, Traditional Chinese Medicine (TCM) provides one of the most sophisticated approaches, with recipes including many herbal drugs to address the multifactorial nature of skin inflammation. The drugs are formulated according to a complex match between patient’s and disease characters and the herbs carefully chosen as to provide opposite characters such as hot, warm, cold, sweet, bitter, pungent, etc. [20,21].

The selection of medicinal plants by traditional Chinese medicine to treat dermatological diseases involving chronic inflammation is done on a complex, multifactorial basis [22]. The diagnosis is usually what traditional Chinese medicine experts refer to as “kidney yin deficiency”, which may be interpreted as a lack of endogenous cortisol. This compound is used clinically in Western medicine in the form of hydrocortisone or prednisone to help control many disorders, including acute inflammations, rheumatoid arthritis, allergies and many eruptic skin diseases. *Angelica* species, traditionally used in the treatment of psoriasis, also act via the “kidney channel” and have been proven to alleviate pain [23]. Since the spleen also plays a major role in immune function, Chinese medicine sometimes calls for the addition of “spleen dampness removing herbs” such as *Poria*, as well as “spleen chi tonics” from species of the genera *Atractylodes* and *Astragalus*. According to traditional Chinese medicine, *Coptis chinensis*, *Paeonia lactiflora*, *Forsythia suspensa* and *Curcuma aromatica* provide analgesic and bacteriostatic properties, along with *Codonopsis pilosula*, which acts as a tonic [24]. Finally, *Phellodendron amurense* and *Scutellaria baicalensis* species are active ingredients of a relatively modern traditional Chinese prescription known as “Three Yellow Cleanser”, which is commonly recommended for many skin conditions [25,26].

### 1.3. Therapeutic Opportunities at the Western–Eastern Interface

The reasons for comparing “Eastern” and “Western” medical frameworks here are to find equivalences between Chinese traditional features/characters for herbal medicines (qualitative adjectives such as cold/warm/neutral, sweet/bitter, etc.) (as presented in Table 1) and “Western pharmacology” biomolecular activities (quantitative data on inhibition of lipoperoxidation and eicosanoids synthesis processes) (as presented in Section 3).

If we “crack” the Traditional Chinese Medicine code, we may find a route to select/identify anti-inflammatory and antioxidant Chinese herbal drugs on the basis of their traditional descriptions, thus maximizing the success of future screenings. Conversely, Chinese researchers and/or practitioners may find a way to add modern molecular meaning to the Traditional classification of such medicinal plants, thus facilitating an integrated approach that may lead to safer, faster and more effective health care [27].

Therefore, our research objectives here are to (1) review the “Western” scientific evidence of these species as anti-inflammatory (eicosanoids inhibition) and antioxidant (enzymatic and non-enzymatic oxidation) to create a qualitative profile of their therapeutic use in skin conditions and then (2) combine experimental (quantitative) biochemical data and traditional Chinese properties with the help of multivariate analysis to unveil links between “Eastern” and “Western” medical frameworks.

## 2. A Focused Review on the Anti-Inflammatory (Eicosanoid Inhibition) and Antioxidant (Lipoperoxidation) Properties of the Selected Medicinal Plants

### 2.1. Methods

Traditional Chinese Medicine may use many herbal drugs as its approach is very multifaceted and the prescriptions are adapted not only to the condition (in this case skin conditions) but also to the patient’s characteristics, making it potentially impossible to cover them all [28]. The plants listed in Table 1 have been the object of intense research by the authors of this review, as well as other research groups, for their topical anti-inflammatory and antioxidant activities [29,30,31,32,33,34,35], thus providing a set of comparable data. The review will revolve around the combined data coming out of the two seminal works of both authors [32,35], complemented and contrasted with all subsequent (and previous if relevant) research done on these herbal drugs in similar or relevant models to the lipoperoxidation framework above discussed.

Literature was sourced from PubMed to ensure pharmacological/medical/clinical relevance by searching by the following combination of keywords [Species name] AND (Cyclooxygenase OR Lipoxygenase OR COX OR LOX OR Lipoperoxidation OR Antioxidant) from 2000 to 2022. Papers with methods or models not relevant or translational to skin conditions and/or treatments at non physiological doses/concentrations were excluded.

### 2.2. Angelica dahurica

Species of the genus *Angelica* (Apiaceae) are used in TCM as ingredients in many medicinal preparations. *A. dahurica*, *A. pubescens* and *A. sinensis* are characterized by containing compounds of the coumarin type.

*A. dahurica* did not attract much attention for its anti-inflammatory or antioxidant activity until Kimura and Okuda [36] mentioned its inhibitory action of histamine release in mice treated with the compound 40/80. The data from our combined studies show that this species was not able to inhibit 5-LOX activity in rat peritoneal PMNs, without being able to determine its action on human platelets due to co-elution problems. The only effect at the cellular level that could be observed was its ability to inhibit the release of elastase as well as its activity, with an IC_50_ of 129 g/µL. It was the only species whose extract was shown to be pro-oxidant in the enzymatic lipid peroxidation model in the CCl_4_/NADPH system, a trend that was repeated in the deoxyribose degradation model by the radical ^•^OH in the absence of ascorbate [32,35]. Further research unveiled that the activity of the plant extract is maximum in its Ethyl acetate fraction, which is endowed with inhibitory effects on LPS-induced TNF-alpha, NO and PGE_2_ production, and expression of iNOS and COX-2 in macrophage through blockade in the phosphorylation of MAPKs, following IκBα degradation and NF-κB activation [37].

**Figure 2:**
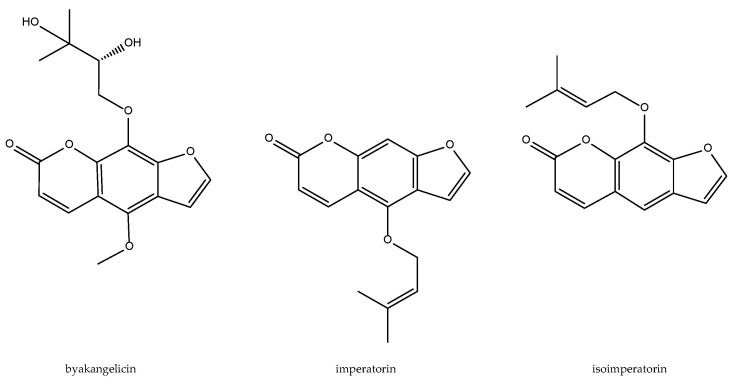
Bioactive anti-inflammatory (eicosanoid inhibition) and antioxidant principles from *A. dahurica.*

A series of bioactive furanocoumarins with inhibitory effects on the arachidonic pathway, namely byakangelicin, imperatorin, and isoimperatorin (Figure 2), have been identified as the anti-inflammatory active principles. Imperatorin showed the most potent inhibitory activity on the LPS-induced PGE_2_ production and expression of COX-2 as well as microsomal prostaglandin E synthase (mPGES) [38]. Byakangelicol, inhibits IL-1β-induced COX-2 expression and PGE_2_ release in human pulmonary epithelial cell line (A549). It is a quite selective COX-2 inhibitor (10–50 µM) when compared to its IC50 > 200 µM for activity and expression of COX-1 in A549 cells; this inhibition may be mediated at least in part by the suppression of NF-kappaB activity [39]. Isoimperatorin exhibits a dual cyclooxygenase-1/2/5-lipoxygenase inhibitory activity measured as PGD_2_ and LTC_4_ biosynthesis in bone marrow-derived mast cells (IC_50_ = 10.7 and 5.7 µM, respectively). The above mentioned bioactive compounds are shown in Figure 2.

### 2.3. Angelica pubescens

Chen et al. reported the anti-inflammatory and analgesic effect of different extracts of *A. pubescens* in in vivo models of formalin- or carrageenan-induced edema [40]. Ko et al. further demonstrated that osthole on platelet aggregation is due to the inhibition of thromboxane formation and phosphoinositides breakdown [41] delayed the aggregation, release of ATP, thrombin and TXB_2_ in isolated and intact rabbit platelets by inhibition of both the TXs synthesis and the inositol pathway. Later, Liu et al. confirmed the inhibitory activity of the dichloromethane extract of *Angelica pubescens* f. *biserrata* on the production of 5-HETE, in intact porcine neutrophils, and PGE_2,_ by microsomes of ram seminal vesicles, both from exogenous ^3^H-AA, isolating and identified the responsible principles as linoleic acid, osthol and osthenol in addition to the polyacetylenes falcarindiol and acetate of 11(S),16(R)-dihydroxyoctadeca-9Z,17-dieno-12,14-diino, all of them with CI_50_ of the order of 20–60 µM [42]. Our own work indicated that, although the ethanolic extract 70% of *A. pubescens* inhibits the production of 5-HETE by 49%, it does not significantly inhibit the total production of the 5-LOX pathway-in rat peritoneal PMNs, since it does not seem to affect the total production of the enzyme, compensating for an increase in LTB_4_ and their isomers. This fact could indicate that the overall effect of the extract lies in an inhibition of the conversion of 5-HPETE, the primary metabolite of 5-LOX, to 5-HETE. The 5-HPETE would more effectively become LTB_4_ than controls. Glutathione peroxidase is the enzyme most directly related to the formation of 5-HETE from 5-HPETE. One can think of a possible action of the extract at this level. Regarding the activity on the COX pathway, measured as production of 12-HHTrE, it can be affirmed that the inhibitory activity of the dichloromethene extract of *A. pubescens* in COX of seminal vesicle microsomes, finds correlation in intact human platelets at the total extract level, where this species is one of the most active (95% inhibition at 200 µg/mL). Since in the methods of antioxidant activity tested, *A. pubescens* was never shown to be active, a mechanism of nonspecific redox inhibition on the above enzymes might be ruled out [32,35]. A more recent paper supports such indirect activity coming from the heteropolysaccharide DF80-2 exhibited antioxidant activity by effectively scavenging hydroxyl radicals and chelating ferrous ions [43].

**Figure 3:**
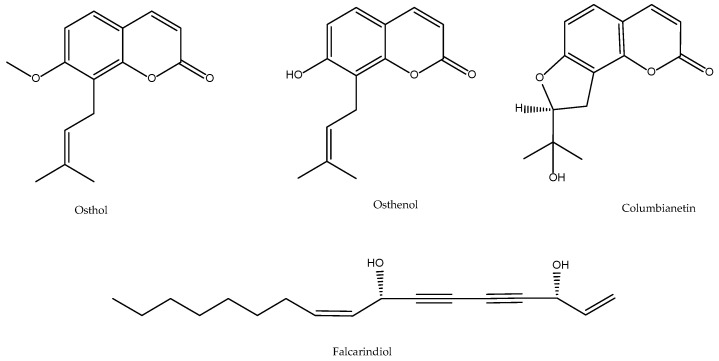
Bioactive anti-inflammatory (eicosanoid inhibition) and antioxidant principles from *A. pubescens.*

Works after 2011 substantiated the importance of columbianetin for the in vivo activities of this TCM drug in suppressing LPS-induced inflammation and apoptosis through the NOD1 pathway [44]. This coumarin is rapidly absorbed when administered orally and has quick clearance and good absolute bioavailability (54–81% for 5–20 mg/kg doses) [45]. The bioavailability of columbianetin is independent of the doses studied. Columbianetin showed dose proportionality over the dose range 5–20 mg/kg. After intestinal absorption this coumarin is likely metabolised by the liver into an array of derivatives as it happens with columbianadin, a closely related compound [46]. Regarding the above-mentioned active principle osthole, it is also active in murine models of neurogenic and inflammatory hyperalgesia by modulation of iNOS, COX-2, and inflammatory cytokines [47] as well as protecting against myocardial ischemia/reperfusion injury [48]. The above-mentioned bioactive compounds are shown in Figure 3.

### 2.4. Angelica sinensis

**Figure 4:**
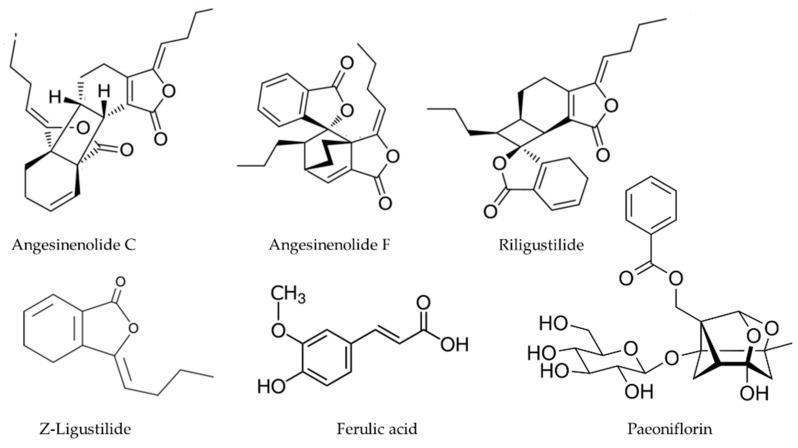
Bioactive anti-inflammatory (eicosanoid inhibition) and antioxidant principles from *A. sinensis.*

*Angelica sinensis* is associated with *Astragalus membranaceus, Cyperus rotundum, Ligusticum chuangxiong* and *Paeonia veitchii* in a formulation called *Danggui,* whose function is to normalize blood rheological values and prevent thrombosis. Ethyl acetate fraction/extracts from this herbal drug have been described as potent anti-inflammatory substances due to the inhibition of pro-inflammatory mediators (NO and PGE_2_) in part via suppression of a signalling pathway such as NF-κB in macrophages [49] as well as rheumatoid synovial fibroblasts [50]. Wang et al. revealed its inhibitory activity of the production of TXA_2_ in porcine pulmonary microsomes [51], and our works demonstrated the total inhibition of 5-HETE production in rat peritoneal PMNs at the dose of 200 µg/mL [32] without any antioxidant activity in our models [35]. This may be in line with previous reports that revealed the effects on lipid peroxidation, the hypoxanthin/XOD system and the hydroxyl radical, after the processing of the drug *radix* *Angelica sinensis,* are highly variable [52]. Perhaps different polarity fractions have different anti-inflammatory and antioxidant profiles in view of report where a supercritical fluid CO_2_ extract attenuated d-galactose-induced liver and kidney impairment in mice by suppressing oxidative stress and Inflammation in terms of MDA levels, enhanced the activities and gene expressions of Cu, Zn-SOD, CAT, and GPx, reduction of iNOS, COX-2, IκBα, p-IκBα, and p65 expression in both hepatic and renal tissues [53]. The effect of this extract and other apolar fractions from *A. sinensis* may be due at least in part by the contribution of its volatile fraction [54] rich in alkylphthalides such as Z-ligustilide n-butylidenephthalide (Figure 4) [55].

Xiet et al. proposed that a polysaccharide is the main effective ingredient of *A. sinensis* and exerts anti-inflammatory effects via down-regulation of COX-1 on LPS-injured PC12 cells [56]. The reader interested on the structure of such class of compound/s can consult the paper by Hou and co-workers on the structure of the main polysaccharides present in this herbal drug [57]. Another class of anti-inflammatory compounds present in *A. sinensis* are dimeric phthalides, some inhibiting COX-2 activity with IC_50_ values as low as 30 μM [58]. *A. sinensis* also contains falcarindiol (Figure 3) [59] and similar polyacetylenes, as well as other well-known eicosanoid biosynthesis inhibitors such as paeoniflorin [60], and ferulic acid [61] (Figure 4).

### 2.5. Astragalus membranaceus

Although *A. membranaceus* has been long studied for its immunomodulatory properties and protective actions of cardiac function [34], there were no references to its anti-inflammatory action until Cuellar et al., demonstrated its activity in several in vivo models of acute edema induced by APD and AA in mouse ear, chronic by multiple applications of TPA, and delayed hypersensitivity induced by oxazolone, without being active in vitro on PLA_2_ of *Naja naja* [31]. The possibility of an inhibition of the lipoxygenase and cyclooxygenase pathways, although not phospholipase, was reinforced after our work: the same extract has been found to inhibit 5-LOX activity in rat peritoneal PMNs and COX-1 activity, although not 12-LOX, in human platelets [32]. According to Wang et al. (1993), *A. membranaceus* would be a better inhibitor of TXA2 production than of PGI2. The extracts reduced the inflammatory response induced by lipopolysaccharide from *E. coli* (LPS) plus interferon-γ (IFN), reducing COX-2 via NF-κB activation in the non-tumorigenic intestinal epithelial cell line (IEC-6) [62].

Despite having a reputation as an antioxidant [28], this plant extract was not active in any of our systems [35]. This discrepancy may be since the studies cited respectively used very high doses of total extract (even 2 mg/mL) or fractions enriched in flavonoids. The total extract was effective at reducing reactive oxygen species (ROS) release though [62].

**Figure 5:**
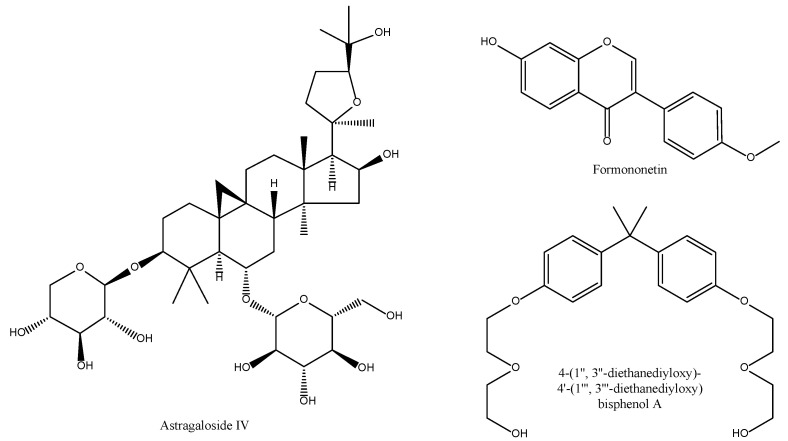
Bioactive anti-inflammatory (eicosanoid inhibition) and antioxidant principles from *A. sinensis.*

It is accepted that the total flavonoids fraction from *A. mebranaceus* reduces both COX-2 mRNA and protein levels [63], Formononetin, a flavonoid present in this Chinese herb (Figure 5), is the main anti-inflammatory and antioxidative principle in different models [64].

The saponin fraction also offers with anti-inflammatory principles such as Astragaloside IV (Figure 5) that prevents UVB-induced oxidative damage in terms of reduced intracellular ROS level and lipid oxidation product malondialdehyde (MDA) content, as well as inflammation by inhibiting TLR4 expression and its downstream signalling molecules (NF-κB, iNOS and COX-2) [65]. Other active principles include bisphenol derivatives (Figure 5) with inhibitory effects on COX-2 mRNA expression at 50 μM [66].

### 2.6. Atratylodes macrocephala

**Figure 6:**
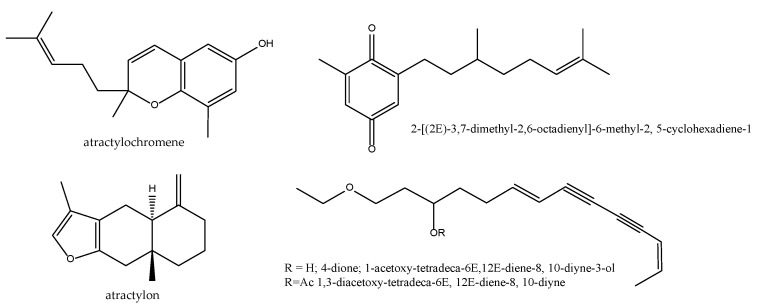
Bioactive anti-inflammatory (eicosanoid inhibition) and antioxidant principles from *Atratylodes macrocephala.*

The data from our previous work indicate the presence of COX-1 specific non-redox inhibitors, which are certainly not particularly active at the total extract level, as is often used in TCM [32]. It is possible that a fractionation results, as in the case of Resch et al. in obtaining sub-extracts with greater activity. These authors demonstrated the inhibitory activity of its hexanic extract at the level of 5-LOX and COX-1 (CI_50_ of 2.9 and 30.5 µg/mL, respectively). Its active ingredients were found to be atractylochromene (Figure 6) and 2-[(2E)-3,7-dimethyl-2,6-octadienyl]-6-methyl-2,5-cyclohexadien-1,4-dione, in addition to a moderate selective activity on 5-LOX of sesquiterpene atractylon (Figure 6) and the coumarin osthol (Figure 2). Preparations based on *Atractylodes* rhizomes are reputed as liver protectors in in vivo models of CCl_4_ toxicity [67]. This activity found no correlation in our works either in enzymatic microsomal lipid peroxidation induced by CCl_4_/NADPH or in the rest of the models tested [35].

Almost two decades after these seminal works, two groups have published new data on this TCM drug. Jeong and co-workers have isolated three polyacetylenes namely 2-[(2*E*)-3,7-dimethyl-2,6-octadienyl]-6-methyl-2, 5-cyclohexadiene-1, 4-dione; 1-acetoxy-tetradeca-6*E*,12*E*-diene-8, 10-diyne-3-ol and 1,3-diacetoxy-tetradeca-6*E*, 12*E*-diene-8, 10-diyne (Figure 6). They showed concentration-dependent inhibitory effects on production of NO and PGE_2_ in lipopolysaccharide (LPS)-activated RAW 264.7 macrophages by suppressing both the protein and mRNA levels via inhibition of nuclear translocation of NF-κB [68]. Wu and co-workers reported last year that the essential oil from this TCM drug containing atractylon (39.22%), β-eudesmol (27.70%), thymol (5.74%), hinesol (5.50%), and 11-isopropylidenetricyclo[4.3.1.1^2,5^]undec-3-en-10-one (4.71%) exhibited strong antioxidant capacities and inhibited NO and PGE_2_ production as well as decreased the transcriptional levels of their originating enzymes in LPS-stimulated RAW264.7 cells [69].

### 2.7. Codonopsis pilosula

According to Wang et al. *Codonopsis. pilosula* is a preferential inhibitor of TXA 2 production over that of PGI_2_ and 6-ketoPGF_1α_ [51,70]. In our own works its CI_50_ resulted higher than the limit of 200 µg/mL, although a tendency was observed to inhibit the 5-LOX and COX-1 pathway with the same efficiency [32]. It did not show any significant activity in peroxidation models or in superoxide radical production at concentrations up to 200 µg/mL either, although it behaved as a pro-oxidant in the deoxyribose degradation model when in the presence of ascorbate [35].

**Figure 7:**
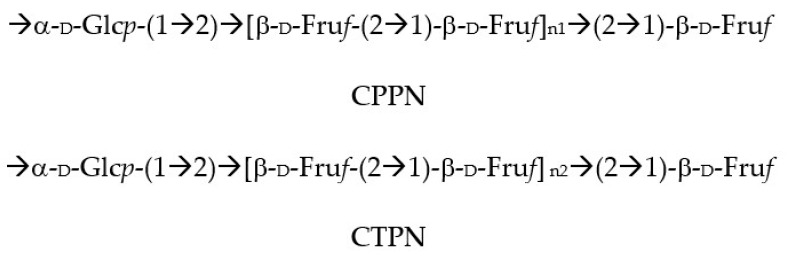
Bioactive anti-inflammatory (eicosanoid inhibition) and antioxidant principles from *Atratylodes macrocephala*. Note n1 < n2.

Efforts to find active small secondary metabolites such as the polyacetylene lobetyolin to antioxidant or anti-inflammatory activities have failed [71]. It was not until recently that more work started to show that the anti-inflammatory and antioxidant effects of the plant may rely on polysaccharides only. On the one hand, the CPP-1 and CTP-1 (Figure 7) can protect IPEC-J2 cells against the H_2_O_2_-induced oxidative stress by up-regulating nuclear factor-erythroid 2-related factor 2 and related genes in IPEC-J2 cells [72]. Furthermore, they increased the total antioxidant capacity, glutathione peroxidase, superoxide dismutase and catalase in the same cells, as well as reducing their levels of MDA [73]. On the other hand, the whole of *Codonopsis pilosula* polysaccharides (CPPS) protect RAW264.7 cells from hydrogen peroxide-induced injury via the Keap1-Nrf2/ARE pathway as well as inhibiting their proinflammatory activities [74,75].

### 2.8. Coptis chinensis

**Figure 8:**
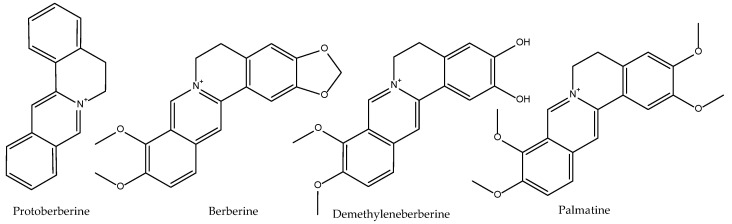
Bioactive anti-inflammatory (eicosanoid inhibition) and antioxidant principles from *Coptis chinensis* and *Phellodendron amurense*.

The rhizome of species of the genus *Coptis* is a reputed remedy in TCM for inflammatory processes. *Coptis chinensis* is usually associated with *Astragalus membranaceus* and *Scutellaria baicalensis* in the medicine called *sanhuang* to treat the so-called Qi Syndrome of venous stasis, showing this preparation inhibitory effects of platelet aggregation [76]. However, only *C. japonica* was studied for its eicosanoid inhibitory until our works [32,35]. Although due to co-elution problems it was not possible to determine the activity on 5-LOX, it was very active inhibiting the production of 12-HHTrE and 12-HETE in human platelets 89% and 70%, respectively, at 200 µg/mL. With these results, the possibility of an action at the PLA 2 level may be a possibility. At the same time Fukuda et al. reported that berberine (Figure 8), a bright yellow isoquinoline alkaloid present in plants of the genera *Berberis* and *Coptis*, effectively inhibits COX-2 transcriptional activity in colon cancer cells in a dose- and time-dependent manner at concentrations higher than 0.3 µM, so it is assumed that *C. chinensis* also acts by an indirect route on the production of eicosanoids [77].

In our hands, the extract exhibited a high inhibitory capacity (83% at 100 µg/mL, CI_50_ = 39 µM) in the CCl_4_/NADPH system of lipid peroxidation as well as 51% in the Fe^3+^-EDTA + H_2_O_2_ system in the presence of ascorbate, being the only active species in this test [35]. Liu and Ng also obtained positive results using the aqueous extract of this herbal drug in models of lipid peroxidation and production of superoxide and hydroxyl radical [78]. These effects are also shown in vivo as recently shown using a murine model of CCl_4_-induced liver injury [79].

Berberine alkaloids (Figure 8) are considered both phytomarkers and active principles of *Coptis* species (Ranunculaceae) but also of the phylogenetically unrelated *Phellodendron amurense* (Rutaceae). All berberine alkaloids suppress—in a variable extend—both the expression and the activity of LOX-5 and COX-2 simultaneously [80]. Berberine (Figure 8) also has anti-inflammatory properties related to its inhibition of NO, Fas, GM-CSF, LIF, LIX, RANTES, and MIP-2 in dsRNA-induced macrophages via the endoplasmic reticulum stress-related calcium-CHOP/STAT pathway [81].

The radical scavenging activity of berberine in the classic models of DPPH^·^ and ABTS^·+^ stable free radical assays was very poor in our hands (IC_50_ > 1000 and 124 µg/mL, respectively) (Data not published). This poor activity is attributed to the lack of phenolic hydroxyl groups to quench the free radicals [82,83]. We also evaluated the ability of the berberine to inhibition non-enzymatic Lipid peroxidation induced by Fe^2+^/ascorbate and CCl_4_/NADPH (enzymatic) in rat liver microsomes, and the IC_50_ were 219 and 105 µg/mL respectively (Data not published). The inhibitory capacity of lipid peroxidation of berberine was also described by other authors [83,84], the increased inhibitory activity of lipid peroxidation in the enzyme system by berberine is attributed in part to the demonstrated inhibitory capacity of different isoforms of CYP450 [85].

### 2.9. Curcuma aromatica

**Figure 9:**
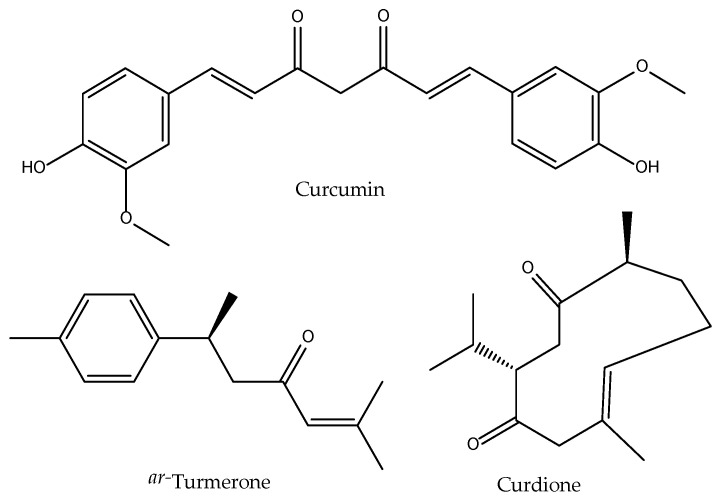
Bioactive anti-inflammatory (eicosanoid inhibition) and antioxidant principles from *Curcuma aromatica*.

In TCM, tubers and dried rhizomes of *C. aromatica* are prescribed, among other things, as analgesics and the anti-inflammatory activity of its essential oil has been studied by Li (1985). However, until our works in the late 1990s few works are found regarding the eicosanoid inhibition properties. Ammon and co-workers described its active principle curcumin—a diarylheptanoid (Figure 9)—as an effective inhibitor of 5-LOX activities in rat peritoneal PMNs, as well as 12-LOX and COX in human platelets, in addition to having a powerful antioxidant effect in in vitro peroxidation models [86]. However, we could not find that the whole extract of the clinically prescribed TCM drug is able to show the same activities in the same models [32,35].

Hundreds of works have reported the topical anti-inflammatory and antioxidant activities of diarylheptanoids—and extracts enriched in these compounds—from *Curcuma sp.* extracts at both experimental and clinical levels [87,88,89,90] and how they regulate both COX and LOX [91] via transcription factors [92]. There is a controversy about the bioavailability of its components that contribute to a huge variability in therapeutic results [93]. The focus on curcuminoids is also shadowing the contribution of other phytochemical present in *Curcuma* sp., and curcumin-free extracts may be also active as recently reported [94].

However, there are only a few works specifically dealing with the effect of *Curcuma aromatica* extracts. Analyses of the data showed that *C. aromatica* consists of various classes of compounds, including alkaloids, flavonoids, curcuminoids, tannins, and terpenoids, that formed the bases of its pharmacological activities [95]. Its content in curcuminoids (curcumin, bis-demethoxycurcumin and demethoxycurcumin) are lower than in *C. longa* [96], thus explaining a lower contribution from this phytochemical class when researching the bioactivities of this herbal medicine. Still it was shown to be more effective reducing the TPA (12-*O*-tetradecanoylphorbol-13-acetate)-induced ear edema in *BALB/c* mice than ibuprofen, an effect that is accompanied by a significant reduction in COX-2 levels in ear tissues [97]. Other constituents that have been related with topical anti-inflammatory activity are those present in the volatile fraction of the tubers/rhizomes of the plant [98]. The sesquiterpenes curdione [99] and *ar*-turmerone (Figure 9) turned out as the major compounds [100] in the essential oils of *C. aromatica* growing in China. Both compounds have the ability to inhibit COX-2 in mouse macrophage RAW 264.7 cells (IC_50_ of 1.1 µM and 24 µM, respectively) [101,102]. This may occur via inhibition of NF-κB activation as shown in breast cancer cells [103]. It also attenuated inflammatory via cytokine expression by inactivating Hedgehog pathway in HaCaT cells [104]. This compound resulted more potent than aspirin at inhibiting platelet aggregation induced by collagen (IC_50_ = 14.4 μM) and arachidonic acid (IC_50_ = 43.6 μM), without any effect on platelet activating factor or thrombin-induced platelet aggregation, thus pointing to a potential direct or indirect inhibition of thromboxane synthesis [105], although COX-1 levels do not change upon *ar*-turmerone treatment in breast cancer cells [103]. Regarding its antioxidant activities, it was shown that extracts of *C. aromatica* effectively protect skin cells from UVA radiations by augmenting their antioxidant defenses [106].

### 2.10. Forsythia suspensa

**Figure 10:**
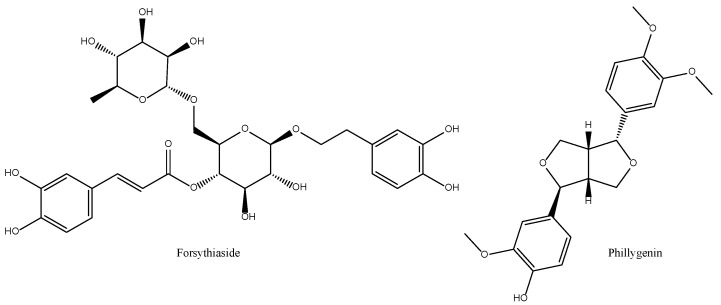
Bioactive anti-inflammatory (eicosanoid inhibition) and antioxidant principles from *Forsythia suspensa*.

The fruits of *F. suspensa* are used in TCM as antipyretics and anti-inflammatories in the treatment of bacterial infections. Kimura and Okuda already described in 1987 that its caffeic acid glycosides are inhibitors of 5-HETE production in rat peritoneal PMNs [36]. Our results expanded on this by showing a concurrent inhibition to LTB_4_ and therefore to the total activity of the enzyme without affecting COX-1 synthesis of 12-HETE in human platelets, thus ruling out any action at the level of PLA_2_ [32]. We later showed that *F.* *suspensa* extract is a potent inhibitor of lipid peroxidation, both enzymatic and non-enzymatic (CI_50_ of 24 and 16.7 µg/mL, respectively) and of the action of the superoxide radical generated by the hypoxanthin/XOD system (CI_50_ = 11.3 µg/mL). However, it enhances the degradation of deoxyribose by the action of the hydroxyl radical generated in the absence of ascorbate [35]. All this pointed towards considering *F. suspensa* as a potentially useful herbal drug at the level of total extract since very marked effects are achieved without having to resort to its fractionation. However, its strong pro-oxidant character in the presence of the hydroxyl radical (system without ascorbate) requires a more careful assessment at the level of cell or organism. There is now some consensus [107,108] in that forsythosides (particularly its A form or forsythiaside) and phillygenin [109,110,111] are among its most important anti-inflammatory and antioxidant active principles (Figure 10).

### 2.11. Lentinus edodes

In our experience, the aqueous extract of *L. edodes* had no activity in either of our eicosanoid pathway (COX-1, COX-2, 5-LOX, 12-LOX, 15-LOX) or antioxidant models [32,35]. Almost at the same time, Sia and Candlish demonstrated that the anti-inflammatory activity of the aqueous extract of this edible fungus lies on the inhibition of interleukin 1 production, without any effect on superoxide radical release in human neutrophils. These authors demonstrated that these effects are due to low molecular molecules rather than macromolecules such as the characteristics polysaccharides (lentinans) present in the mushroom [112].

**Figure 11:**
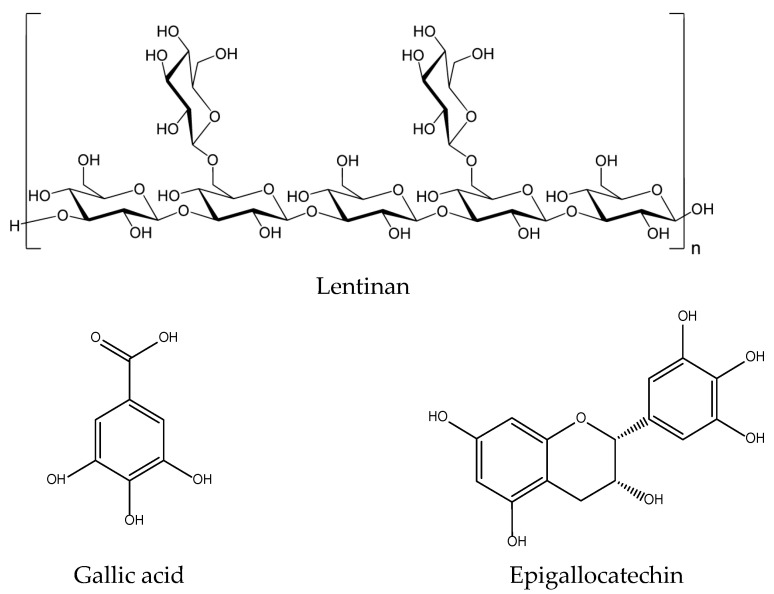
Bioactive anti-inflammatory (eicosanoid inhibition) and antioxidant principles from *Lentinus edodes*.

Small phenolic secondary metabolites have been described in *L. edodes* including precursors of tannins such as gallic acid and epigallocatechin (>50 mg/kg) (Figure 11) and in lower quantity flavonoids such as isoquercetin, kaempferol and eriodictyol levels (<50 mg/kg) [113]. These compounds may justify potential anti-inflammatory and antioxidant activities in the models mentioned above only if concentrated at pharmacological-relevant levels [114].

Lentinan (Figure 11), extracted from its fruiting body, has clinically significant anticancer, antibacterial, antiviral, and anticoagulant effects. There is a report on its preventive effects on skin oxidative damage by H_2_O_2_, reduction MDA formation, and increased SOD activity in HaCat cells [115] as well as on the inhibition of the production of pro-inflammatory cytokines, including IL-1β, TNF-α, IL-8 and the secretion of PGE_2_ and NO, by reducing the expression of COX-2 and iNOS in AGE-challenged chondrocytes [116].

It could be said that the main clinical interest of this species in the treatment of pathologies that occur with inflammatory processes would derive from indirect effects at the level of the microbiota [117] or immunological level [118] and not to direct inhibitory actions on the LOX, COX or the production of free radicals in proinflammatory cells.

### 2.12. Paeonia lactiflora

**Figure 12:**
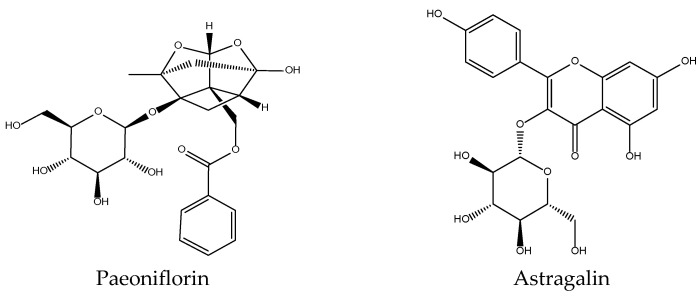
Bioactive anti-inflammatory (eicosanoid inhibition) and antioxidant principles from *Forsythia suspensa*.

*P. lactiflora* is a Ranunculaceae with a reputation for analgesic and bacteriostatic effects. Preliminary work demonstrated the inhibitory activity of one of its components, paeoniflorin, in platelet aggregation models [24]. Our results supported the existence of a specific action at the platelet level, since the total extract of this drug inhibited 70% the production of 12-HHTrE without altering the levels of 12-HETE, LTB_4_ or 5-HETE [32]. In the free radical generation tests, pro-oxidant was shown in the Fe^3+^-EDTA + H_2_O_2_ system without ascorbate [35]. These data would justify the use of the total extract as an analgesic, since it inhibits COX-1 activity in vitro, although in vivo this effect may also be due in part to the central nervous system depressant action of paeoniflorin (Figure 12) [24]. Little additional work has been done in this direction apart from the confirmation of anti-inflammatory effects of this compound in human dermal microvascular endothelial cells cancer cells by blocking nuclear factor-κB and ERK pathway [119]. Its antioxidant effects in UVA-induced damage in human dermal fibroblasts in terms of reduction of the ROS and MDA levels is due to the inhibition of the Nrf2/HO-1/NQ-O1 signalling pathway [120]. Astragalin (Figure 12) is another secondary metabolite present in this herbal drug that has been studied [121].

### 2.13. Phellodendron amurense

This rutaceae is, like *Coptis chinensis,* rich in alkaloids of the berberine type (berberine, palmatine, etc.) (Figure 8). Our works described the in vitro actions on the production of eicosanoids and free radicals by its total extract: although its action on LTB_4_ could not be quantified, a total inhibition of 5-HETE production was found, as well as an inhibition of 86% and 65% in the production of 12-HHTrE and 12-HETE, respectively at 200 µg/mL [32]. With these data, an effect at the level of PLA_2_ cannot be ruled out. In the enzymatic lipid peroxidation model, it obtained an IC_50_ = 21.6 µg/mL, not being particularly active in any of the other methods tested [35].

Müller and Ziereis could not demonstrate any significant activity of berberine on 5-LOX [122]. The bioactive alkaloids identified from this herbal drug (Figure 8) suppress the expression of LOX-5 and COX-2 simultaneously in rat cell and models of Bening Prostate Hyperplasia. In particular, protoberberine and demethyleneberberine were found to exhibit strong direct inhibitory activities against both LOX-5 and COX-2 enzymes, whilst palmatine and berberine showed moderate inhibitory activities only. Molecular docking analysis confirmed that demethyleneberberine could directly interact well with LOX-5/COX-2 [80].

### 2.14. Poria cocos

*Poria cocos* extracts inhibited PLA_2_-induced mouse paw edema by both the oral and parenteral routes [123]. Subsequent work led to the isolation of lanosthane-type anti-inflammatory principles with interesting PLA_2_ inhibitory activity from *Naja naja* in vitro and in vivo [29,30]. We described how this very same extract inhibits both the production of 5-LOX metabolites in rat peritoneal PMNs and 12-HHTrE and 12-HETE in human platelets. With very similar percentages of inhibition thus supporting an activity at PLA_2_ level_,_ in line with works using by an in vitro polarographic, we also described for the first time the antioxidant activity of extracts from this fungus, which inhibited 43% the degradation of deoxyribose by the hydroxyl radical generated by the Fe^3+^-EDTA + H_2_O_2_ and ascorbate system. In this model, no other extract showed an effectiveness greater than 40%, except *Coptis chinensis* [35]. Little additional work has been done to unravel these activities. There is only one report on its anti-skin aging effects via activation of the Nrf2-antioxidant mechanism in human dermal fibroblasts [124].

**Figure 13:**
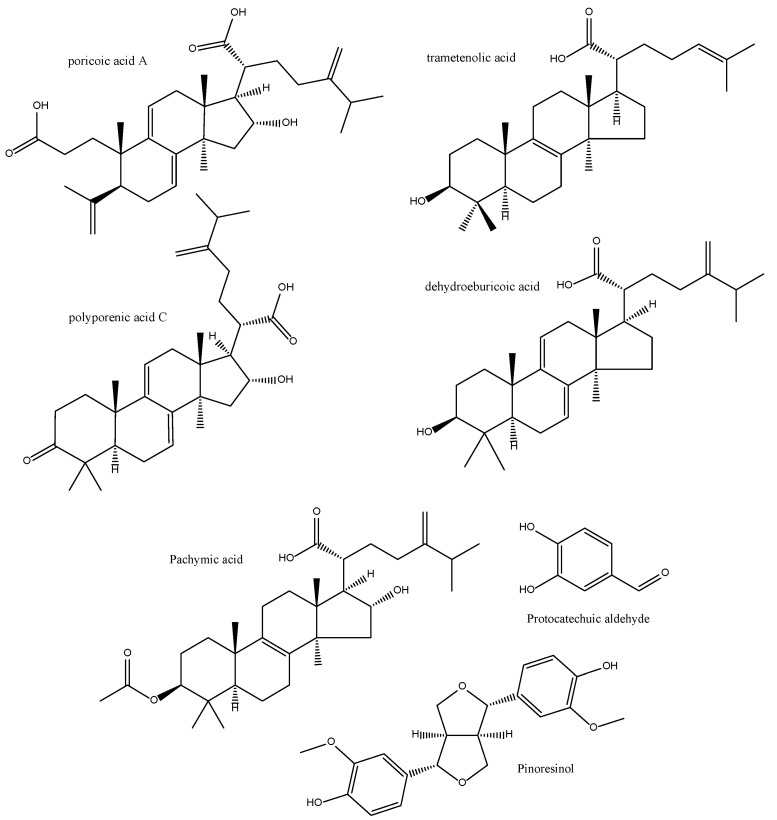
Bioactive anti-inflammatory (eicosanoid inhibition) and antioxidant principles from *Poria cocos*.

The anti-inflammatory principles of the EtOH extract of the sclerotia of *P. cocos* after bioassay-guided fractionation using LPS-stimulated Raw264.7 cells, include triterpenoids (such as poricoic acid A, polyporenic acid C, trametenolic acid and dehydroeburicoic acid) as well as phenolics (pinoresinol and protocatechualdehyde) (Figure 13). They all have inhibitory effects on the production of NO, PGE2 and the expression of iNOS) and COX-2 [125]. Pachymic acid (Figure 13), another characteristic lanostane-type triterpenoid from *Poria cocos*, exerts anti-inflammatory and antioxidant effects in mice kidneys by increasing glutathione expression, decreasing MDA and COX-2 levels and increasing the expression levels of several NRF2 signaling pathway proteins [126]. Similarly, dehydrotrametenolic acid (Figure 13) can activate AP-1 and NF-κB transcriptional factors in human keratinocyte cell line HaCaT cells which may in turn modulate the arachidonate pathway [127]. The free radical scavenging activities of lanostanes are not very prominent, though [128].

As in the case of other higher fungi such as *Lentinus edodes*, polysaccharides are prominent in the chemical make-up of *P. cocos* aqueous extracts. These have been described as having in vitro antioxidant activities on the basis of DPPH radical, hydroxyl radical, reducing power and metal chelating ability [129]. Strikingly, such compounds have been reported to be pro-inflammatory as they can stimulate macrophages to express iNOS gene through the activation of NF-κB/Rel and interleukins, interferon and TNF through TLR4/TRAF6/NF-κB signalling both in vitro and in vivo [130]. Extracts containing lanostane triterpenoids also enhance non-specific (innate) immunity though activating natural killer cells and regulating interferon and interleukin synthesis in T-helper cells 1 and 2, respectively, thus modulating the cellular immune response [131]. Therefore, the balance of pro- and anti-inflammatory effects of *P. cocos* extracts may hugely vary depending on the polarity of the solvents and methods of extraction. The anti-inflammatory and antioxidant overall effects of truly whole extracts may be extremely difficult to predict and even turn out being pro-inflammatory.

### 2.15. Rehmannia glutinosa

Early works reported its in vivo effects on the platelet [132] and the action at the immunomodulatory level of its polysaccharides as shown by a pronounced anti-complementary activity [133]. In our hands the extract of *R. glutinosa* did not show any significant anti-inflammatory or antioxidant effects [32,35]. The presence of iridoids, especially catalpol, could suppose potential anti-inflammatory and antioxidant activities via modulation of pro-inflammatory cytokines, as seen in Caco-2 cells [134] and may contribute to skin healing due to the effect of acteoside, a phenylethanoid glycoside isolated from the leaves of this herbal drug, activating the expression of matrix metalloproteinases (MMPs) in normal human dermal fibroblasts [135].

### 2.16. Scutellaria baicalensis

**Figure 14:**
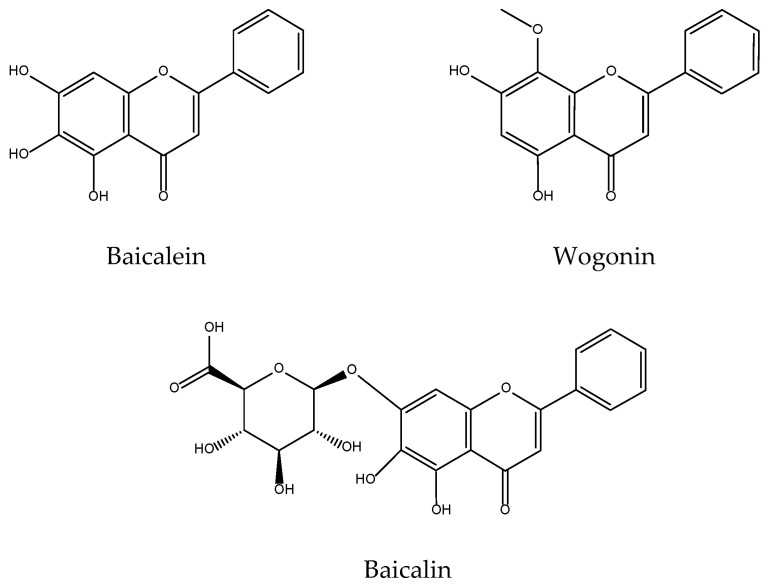
Bioactive anti-inflammatory (eicosanoid inhibition) and antioxidant principles from *Scutellaria baicalensis*.

The pharmacological properties of the genus *Scutellaria,* and in particular *S. baicalensis,* have aroused great interest in the scientific community, an interest that is reflected in the large amount of earlier work done on its antioxidant and anti-inflammatory activities, especially of three of its main components: baicalein, baicalin, and wogonin (Figure 14) [136,137].

Although in our study *S. baicalensis* interfered with the quantification of arachidonic acid metabolites, an absence of 5-HETE production was observed. Although no previous work has been found reporting this effect, Butenko et al. described the inhibitory action of baicalin in the production of LTC_4_ in peritoneal macrophages of rats [136] and You et al. in 12-LOX without affecting COX activity [137].

Our works on the antioxidant activity of *S. baicalensis* extracts corroborate the interest of the total extract as an inhibitor of lipid peroxidation (CI_50_ of 5.3 µg/mL and 13.6 µg/mL in the enzymatic and non-enzymatic systems, respectively). It could not be tested in the hypoxanthin/XOD system since the total extract interferes with XOD activity, probably due to its content in baicalein, baicalin and wogonin, which according to Chang et al. are inhibitors of this activity [138]. In the hydroxyl radical generator system Fe^3+^-EDTA+H_2_O_2_ with and without ascorbate, the total extract of *S. baicalensis* was shown to be extremely pro-oxidizing, promoting the degradation of deoxyribose by 852% and 157%, respectively. This surprising fact, given that baicalein and baicalin are inhibitors of this effect [139] could only be explained by the fact that another characteristic flavonoid, escutelarin, is pro-oxidant [140], an effect that prevails in the total extract. This fact emphasizes the importance of testing the total extracts, as they are used ethnopharmacologically, instead of their components or fractions, since the observed effects can be radically different.

Later research strengthens baicalein as a protective agent for skin cells from the oxidative stress caused by H_2_O_2_ through activation of Nrf2 signalling pathway [141,142]. It also protects human keratinocytes from UV-induced ROS-mediated damage [143,144] thus explaining previously claims of the UV protection conferred by *S. baicalensis* crude and flavonoid-enriched extracts [145]. Effects of both baicalein and wogonin (Figure 14) in acute UVB-irradiated cells involve reduced levels of COX-2 [146], although it has been observed that high doses may slightly induce COX-1 mRNA, although eventually a decrease of PGE_2_ is always observed in wogonin-treated mice [147].

## 3. Unveiling Links between Traditional Chinese Plant Characters and Quantitative Antioxidant/Eicosanoid Inhibitory Activities of the Extracts

Our aim is to investigate now if the data point out to any link/s between the inhibitory properties of lipid peroxidation and eicosanoid biosynthesis in medicinal plants and the properties/characters that they are assigned to these herbal drugs by TCM doctors.

### 3.1. Data Sourcing

Most of the biochemical and pharmacological activities of the TCM drugs listed in Table 1 were published in two articles [32,35]. The origin of the plants and the extracts were maintained, thus ensuring that both sets of antioxidant and eicosanoid inhibition data are comparable. Where gaps existed, we filled with results from the literature using similar substances if available. All the raw data were normalised to percentage of inhibition of the biochemical or pharmacological endpoint (0% maximum inhibitory effect–100% minimum inhibitory effect, using MS Excel (Microsoft, Redmond, Washington). The data are summarised in Table 2. A three-dimensional vector positioned each herbal drug in an “antioxidant” space, defined by the values of the three antioxidant tests: (LNE) Lipid Non-Enzymatic Peroxidation; (LE) Lipid Enzymatic Peroxidation; (XO) Xanthine Oxidase. The magnitude of each 3D “antioxidant” vector was calculated (α). Another bi-dimensional vector positioned each herbal drug in an “anti-inflammatory” space, defined by the values of the 5-LOX and COX-1 eicosanoid biosynthesis tests. The magnitude of each 2D vector was calculated (β). Another three-dimensional vector positioned each herbal drug in a wider “anti-inflammatory” space, defined by the values of the 5-LOX, 12-LOX and COX-1 eicosanoid biosynthesis tests eicosanoid biosynthesis tests. The magnitude of each 3D vector was calculated (χ). The α and β values positioned each extract in a bidimensional space. These set was subject to cluster analyses by *k*-means. In a separate analysis, the α and χ values positioned each extract in a bidimensional space (antioxidant activity in *X* axis vs. eicosanoids inhibition in *Y* axis), and these sets were subject to cluster analyses by *k*-means. All *k-*means clustering was performed with SPSS 19 (IBM, Armonk, NY, USA).

### 3.2. Results and Discussion

**Figure 15:**
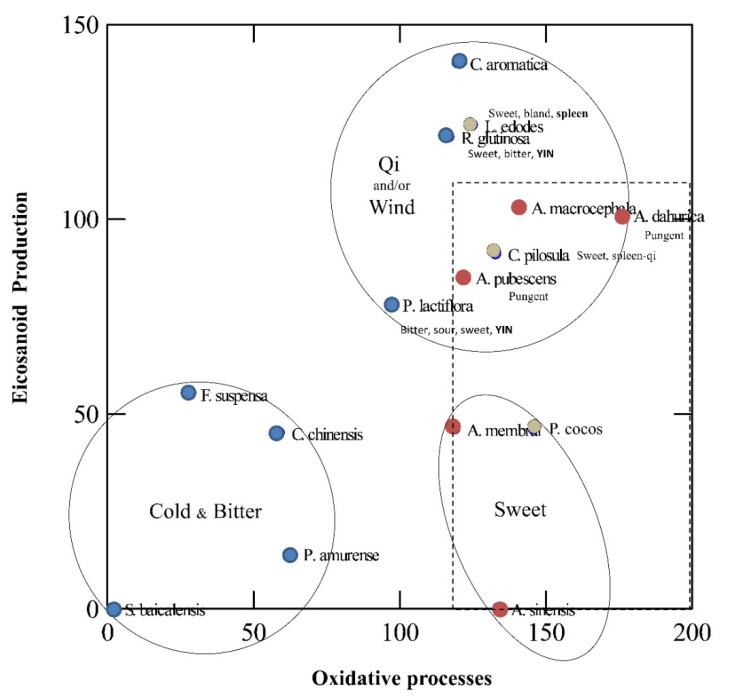
Scatterplot of the lipoperoxidative activity (*X* axis, α values) vs. COX-1/5-LOX inhibition (*Y* axis, β values). Blue spots: Cold Herbs; Red spots: Warm/Hot Herbs; Grey spots: Neutral Herbs.

When all selected TCM drugs were analysed for the relationship between their combined inhibitory properties on COX-1/2 and 5-LOX versus their combined antioxidant effects, three clusters were identified (Figure 15). The first one contains 4 plants characterised by high inhibitory activity of all the biochemical parameters. A second cluster was interpreted as plants with high inhibitory properties on eicosanoids release but mild/low antioxidant properties, and a third group of plants had mild/low activities on both parameters.

When overlapping TCM characters for each herbal drug, we could observe that the first cluster is coherent with some phytochemical traits as well as therapeutic uses in TCM (Table 1). Interestingly, all are considered “Bitter and cold”. Generally speaking, Chinese cleansing herbs are considered bitter herbs with a “cold property”. Phellodendron (*Huang Bai*), and Skullcap (*Huang Qin*) are the constituents of a popular herbal formulas used for a variety of skin disorders, the “Three Yellow Cleanser” (*San Huang Xi Ji*, where *Huang* means yellow), together with Rhubarb (*Dai Huang*) and Sophora (*Ku Shen*). Furthermore, Phellodendron and Coptis [25,26] share the same chemistry based on berberine alkaloids. The interchangeability of Phellodendron (“Drains Fire and relieves Fire toxicity”) and Coptis is known in both traditional and local medicinal systems in China as “Using different plants as the same herbal medicine” (使用不同的植物作为同一种药草). Features to identify *Huang-lian* are “yellow and bitter”, and chemically speaking this is strongly related to the presence of berberine type alkaloids. Theoretically, *Scutellaria, Berberis,* and *Thalictrum* species could be indistinctly used as *Huang-lian* (黄连) the common name for *Rhizoma coptidis*. However, in a study of the local medicine in NW Yunnan, the authors found that other herbs with different chemistry but overall same pharmacological features such as, *Scutellaria* spp (Huang-Qin, which also “Drains Fire and detoxifies”) were used as *Huang-lian* [151], thus supporting our results. The presence of *F. suspensa* in this cluster is surprising, as it is chemically very different but highly reputed for abscess and sores, sore throat, scrofula and subcutaneous nodules [24]. In the light of our review (see Section 2.10) not much “Western” science is available for this otherwise promising “anti-inflammatory and antioxidant” herbal drug.

The second cluster contains a mix of two hot herbs and one neutral fungi, all sweet in nature. They can strongly inhibit the synthesis of eicosanoids but show mild antioxidant activity. Therefore, these herbs may mimic NSAIDs or steroids activity. Indeed *A. membranaceus* and *P.cocos* contain steroidal-like compounds, namely astragalosides and lanostanes as reviewed in Section 2.5 and Section 2.14, respectively. *A. dahurica* is clearly separated from its two congeners, *A. sinensis* and *A. pubescens*. Interestingly, the “expel wind” action is almost exclusive of the two furanocoumarins containing *Angelica* species (*A. dahurica,* and *A. pubescens*) and differentiates them from *A. sinensis,* which “tonifies blood” but is not a significant source for such photodynamic compounds as per a recent review [152].

The third cluster is composed of a mix of warm, cold, and neutral drugs. However, the warm character seems to be confined to a particular region of the biochemical space (delimitated by a dashed red square) close to the second cluster. The differential trait here seems to be either tonifying/moving Qi and/or “expelling wind” actions as well as protecting the spleen and/or liver. The action on Qi seems to correlate with immunomodulation or protection of internal organs. The unprocessed *R. glutinosa* is a cortisol-like substance, which has the advantage of not suppressing, but rather enhancing, the immune system in many cases [23], and our review supports these immune effects via cytokines (see Section 2.15). The fungi *L. edodes* that acts as a “liver-enhancing” herbal medicine, thus protecting the liver from damage associated with autoimmunity, inflammation, oxidation, and infection [153], and *C. aromatica* that similarly cleans the liver and the blood (Table 1) seems to be linked to the Western “detoxification” concept. The activities of *A. macrocephala* and *P. lactiflora* target spleen and liver (Table 1), thus implying both detoxification and immunomodulatory effects.

**Figure 16:**
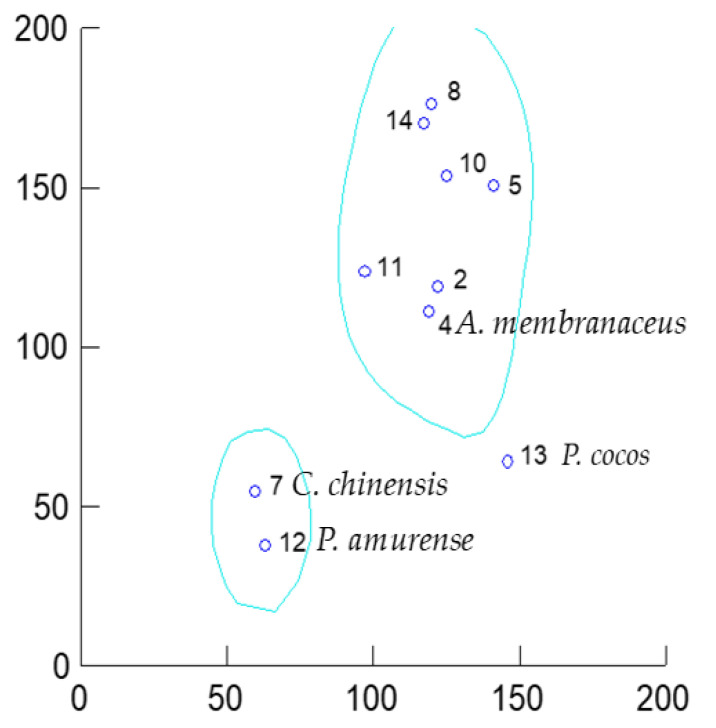
Scatterplot of the antioxidant activity (α values in *X* axis) vs. eicosanoids inhibition (χ values, *Y* axis) of the extracts. Numbers denote the species as per Table 2. Only key species for discussion are named here.

When a reduced set of TCM drugs were analysed for the relationship between the combined inhibitory properties on COX-1, 5-LOX and 12-LOX vs. their combined antioxidant effects, three clusters were again identified (Figure 16).

The inclusion of the inhibition of 12-LOX inhibition—although restricting the dataset—points towards a “selective” influence of this eicosanoid pathway within the skin lipoperoxidation model. 12(S)-HETE is present in psoriatic scales [1]. Interestingly, human platelets produce 12(S)-HHTrE and 12(S)-HETE from the COX-1 and 12-LOX pathways, respectively, after stimulation with Ca++ and ionophore A23187. Therefore, its use as in vitro screening for anti-psoriatic drugs is relevant since in psoriatic epidermis only the platelet-type 12-LOX is detectable [154]. The lack of inhibition of 12-LOX also rules out any impairment of the release of endogenous arachidonic acid from the membranes by phospholipase A2 since endogenous arachidonate is available to 12-LOX. The two berberine alkaloid-containing herbal drugs, *C. chinensis* and *P. amurense,* remain in the Cold/Bitter cluster with very similar and relatively low IC_50_s for this enzyme, whilst *F. suspensa* -which fails to be active at this level- and *S. baicalensis* -for which no data on 12-LOX could be found- are now out of the picture. The Sweet-Hot/Warm cluster now contains the fungi *P. cocos* only.

## 4. Conclusions

We here present a thorough review of the eicosanoid inhibitory activities of important Chinese herbal drugs, as well as a biochemical explanation to some of the characters and actions of TCM drugs used—among other conditions—in skin diseases. Lipid peroxidation and eicosanoids production are intimately linked, and our cluster analysis unveiled how cleansing herbs of bitter and cold nature acting through removal of toxins—such as *P. amurense, Coptis chinensis, S. baicalensis* and *F. suspensa*—are highly correlated with strong inhibition of both lipid peroxidation and eicosanoids production. Sweet drugs—such as *A. membranaceus, A. sinensis* and *P. cocos—*act through a specific inhibition of the eicosanoids production. The therapeutic value of the remaining drugs with low antioxidant or anti-inflammatory activity—seems to be based on their actions on the Qi with the exception of furanocoumarin containing herbs—*A. dahurica* and *A. pubescens—*which “expel wind”.

A further observation from our results is that the drugs present in the highly active “Cleansing herbs” cluster are commonly used for skin conditions and may be bioequivalents (=interchangeable) thus supporting the special concept of Traditional Chinese Medicine called “Multisource” of herb (多基源). The inclusion of 12-LOX inhibition did not fundamentally change the clusters but pointed towards plants that may be more active in chronic skin conditions such as psoriasis.

## Figures and Tables

**Table 1:** Scientific, pharmacopeial and Chinese names of the selected herbal drugs and their properties and actions according to the traditional Chinese medicinal system [20,21].

TCM Drug Botanical Species Chinese Name/Other Names	Properties Meridians	Actions
*Radix Angelica dahurica* *Angelica dahurica* Fisch. ex Hoffm. Bai Zhi/Chinese angelica	Pungent and warm. Lung, stomach and large intestine.	Expel wind and release exterior, alleviate pain, relieve stuffy nose, dry dampness, and stop leucorrhoea.
*Radix Angelica pubescens* *Angelica pubescens* Franch. Du Huo/Shishiudo	Pungent, bitter, slightly warm. Liver, kidney, and lung.	Dispel wind-damp, alleviate pain, release exterior.
*Radix Angelica sinensis* *Angelica sinensis* (Oliv.) Diels Dang Gui/Female ginseng	Sweet, pungent, warm. Heart and liver.	Tonify blood, activate blood, alleviate pain, regulate menstruation, and moisten intestines.
*Radix Astragali* *Astragalus membranaceus* (Fisch.) Bunge (Now *A. propinquus* Schischkin) Huang Qi/Mongolian milkvetch	Sweet, warm. Lung and spleen.	Tonify qi, raise yang, tonify defensive aspect to secure superficial, relieve edema through diuretic, dispel toxin to promote skin generation, nourish blood.
*Atractylodis macrocephalae rhizome* *Atractylodes macrocephala* Koidz. Bai Zhu	Sweet, bitter, warm. spleen and stomach.	Tonify spleen qi, dry dampness, induce diuresis, arrest sweating and prevent abortion.
*Radix Codonopsis* *Codonopsis pilosula* Franch. Dang Shen	Sweet, neutral. Lung and spleen.	Invigorate lung-qi and spleen-qi, nourish blood, and promote the generation of body fluid.
*Rhizoma Coptidis* *Coptis chinensis* Franch. Huang Lian	Bitter, cold. Heart, stomach, Large intestine and liver.	Clear heat and dry dampness, purge fire and relieve toxicity.
*Radix Curcumae* *Curcuma aromatica* Salisb. Yu Jin/Turmeric	Pungent, bitter, cold. Liver, gallbladder and heart.	Activate blood and alleviate pain, move qi and relieve depression, clear heat and cool blood, promote excretion or bile and remove jaundice.
*Fructus Forsythiae* *Forsythia suspensa* (Thunb.) Vahl Lian Qiao/Weeping forsythia	Bitter, slightly pungent, cold. Lung, heart and small intestine.	Clear heat and remove toxicity, disperse wind-heat, clear heart-heat.
*Lentinus edodes* *Lentinus edodes* (Berk.) Pegler Xianggu/Oakwood mushroom	Sweet, neutral. liver and stomach.	Tonify deficiency, strengthen the spleen, stimulate the appetite, expel wind, and promote eruption, resolve phlegm and regulate the flow of qi, remove toxicity and treat cancer.
*Radix Paeoniae Alba* *Paeonia lactiflora* Pall. Bai Shao (Chi Shao)/Chinese peony	Bitter, sour, sweet, slightly cold. Spleen and liver.	Tonify blood, astringe yin to check sweating, emolliate liver to alleviate pain, calm and suppress liver yang.
*Phellodendri Amurensis Cortex* *Phellodendron amurense* Rupr. Huang Bo/Amur cork tree	Bitter, cold. Liver, gallbladder, Large intestine, kidney and bladder.	Clear heat and dry dampness, purge fire and remove toxicity, subdue deficiency heat.
*Poria* *Poria cocos* F.A.Wolf Fu Ling/Poria	Sweet, bland, neutral. Heart, spleen, and kidney.	Induce diuresis and drain dampness, invigorate spleen, and induce tranquilization.
Radix Rehmanniae *Rehmannia glutinosa* (Gaertn.) DC. Di Huang/Chinese Foxglove	Sweet, bitter, cold. Heart, liver, stomach and kidney.	Clear heat and cool blood, stop bleeding, nourish yin
*Radix Scutellariae* *Scutellaria baicalensis* Georgi Huang Qin/Skullcap	Bitter, cold. Lung, stomach, gallbladder, large intestine or bladder.	Clear heat and dry dampness, purge fire and relieve toxicity, cool blood, and stop bleeding.

**Table 2:** Biochemical activities (percentage of inhibition of the measured endpoint) of the selected TCM herbal drugs and magnitude of the resulting vectors. Warm colours denote high inhibitory activity whilst cold colours the opposite.

TCM Drug		Lipoperoxidation [35]				Eicosanoids [32]				
		LNE	LE	XO	α	5LO	COX1	β	12LO	χ
**1.**	*A. dahurica*	94	149	74	**191**	98	25 ^a^	**101**	-	**-**
**2.**	*A. pubescens*	88	85	73	**122**	85	5	**85**	83	**119**
**3.**	*A. sinensis*	95	94	90	**133**	0	0 ^b^	**0**	-	**-**
**4.**	*A. membranaceus*	89	79	116	**119**	37	28	**46**	101	**111**
**5.**	*A. macrocephala*	91	108	89	**141**	86	56	**103**	110	**150**
**6.**	*C. pilosula*	94	94	99	**132**	62	66	**91**	-	**-**
**7.**	*C. chinensis*	57	17	79	**60**	44	11	**45**	30	**54**
**8.**	*C. aromatica*	92	77	54	**120**	86	111	**140**	106	**176**
**9.**	*F. suspensa*	3	28	20	**28**	24	50 ^c^	**55**	119	**131**
**10.**	*L. edodes*	92	85	88	**125**	104	67	**124**	91	**154**
**11.**	*P. lactiflora*	74	63	56	**97**	72	30	**78**	96	**124**
**12.**	*P. amurense*	60	20	85	**63**	0	14	**14**	35	**38**
**13.**	*P. cocos*	94	112	75	**146**	39	25	**46**	44	**64**
**14.**	*R. glutinosa*	92	73	79	**117**	104	61	**121**	120	**170**
**15.**	*S. baicalensis*	2	1	-	**2**	0 ^d^	0 ^e^	**0**	-	**-**

(LNE) Lipid Non-Enzymatic Peroxidation; (LE) Lipid Enzymatic Peroxidation; (XO) Xanthine Oxidase; (5LO) 5-Lipoxigenase; (COX-1) Cycloxigenase-1; (12LO) 12-Lipoxigenase; (α) Magnitude of the vector defined by the inhibition of the antioxidant models; (β) Magnitude for the vector defined by the inhibition of the COX-1/2 and 5-LOX; (χ) Module for the vector defined by the inhibition of all LOX and COX activities; (^a^) COX-2 Inhibition value extracted from Hwang et al. [148]; (^b^) PGE2 inhibition value extracted from Chao et al. [49]; (^c^) PGE2 inhibition value extracted from et Kim et al. [149]; (^d^) Leukotriene inhibition value extrapolated from Kim et al. [149]; (^e^) PGE2 inhibition value extrapolated from Ye et al. [150].
